# Automated Periotome versus Conventional Periotome in Intra-Alveolar Extraction of Sheep Jaw: An In Vitro Study

**DOI:** 10.1155/2022/5058606

**Published:** 2022-07-13

**Authors:** Sam Thomas Kuriadom, Sarmad Al-Chalabi, Karrar M. H. Hadi, Ashraf M. Ishbair

**Affiliations:** ^1^Department of Clinical Sciences, College of Dentistry, Ajman University, Ajman, UAE; ^2^Center of Medical and Bio-allied Health Sciences Research, Ajman University, Ajman, UAE; ^3^Bright Smile Dental Clinic, Al Ain, UAE; ^4^College of Dentistry, Ajman University, Ajman, UAE; ^5^Hakeem Dental Center, Abu Dhabi, UAE

## Abstract

**Background:**

Atraumatic dental extraction is the way forward in modern dentistry. This study aims to compare the effectiveness of automated periotome with conventional periotome with regard to operating time, postoperative gingival laceration, and bone and tooth structure fractures.

**Methods:**

This is an in vitro study of forty posterior teeth of sheep mandibles. Ten sound healthy mandibles were selected, and each mandible was then divided into two quadrants with two teeth in each quadrant. Teeth were then extracted by conventional periotome for the first group (one quadrant) and by automated periotome for the second group (other quadrants). A statistically significant *P* value is set at below 0.05 with a 95% confidence interval.

**Results:**

No bone fracture was seen in any of the cases of automated periotome with a significance of 0.004 when compared to the fractures seen in seven cases in the conventional group. Whereas comparing the other parameters among the different groups did not show any significant difference.

**Conclusion:**

It is worthwhile to use the automated periotome in simple extractions, especially when implants are considered in the treatment plan.

## 1. Introduction

Dental elevators have endured the test of time and are commonly used during various oral surgical procedures. Over the years, to improve their efficiency, they have evolved with regard to shapes, names, and even their material characteristics [1]. Elevators were, perhaps, introduced into general dental practice for the first time by Thomas Bell. Initially, these instruments were used for the removal of lower third molars [2].

Dental schools have traditionally been teaching dental extractions in the same way for many years. The focus of attention is getting the tooth out with little attention to supporting tissues [3]. Dental extraction, depending on the difficulty, traditionally involves detaching the mucogingival collar around the tooth and even occasionally a bit of mucoperiosteum. Sometimes, the extraction causes alveolar bone damage and soft tissue injury [4]. The traditional method of extraction by exerting force on the alveolar bone using dental elevators and sometimes even grasping a small portion of the alveolar bone with the extraction forceps can lead to postoperative deformation of the dentoalveolar housing. Eventually, this causes ridge defects and the surgical insertion of dental implants problematic [5].

In addition, the habitual elevation of the mucoperiosteum by many surgeons leads to compromise in the blood flow to the alveolar bone from the periosteum. This can cause a deficit of marginal alveolus even after relatively simple or minimally invasive extraction. Therefore, it is vital to maintain the alveolar bone. Maintaining the alveolar bone also allows for better functional and cosmetic restorations, especially with dental implants [5]. For that reason, following dental extraction, alveolar ridge preservation, with the application of different biomaterials, is the most common procedure aiming to control crestal bone resorption, which could decrease the necessity of advanced regenerative procedures prior to dental implant placement [6].

Implant stability is one of the most critical goals of implant rehabilitation [7]. Several factors influence primary implant stability (PS), including implant shape, surgical method, and bone quantity and quality [8, 9]. To enable an immediate-loading protocol and achieve long-term stability, a good PS is essential [10]. Furthermore, in preserving good bone quantity and quality, the patient would have more than one treatment option in restoring missing teeth where in cases a conventional complete denture is deemed inappropriate in terms of having an increased risk of progressive bone loss, lower stability and retention, loss of periodontal proprioception, and low masticatory efficiency [11]. Also, in cases with syndromes such as the oral-digital-facial syndrome (OFD), it is beneficial to the patient to remove all the supernumerary teeth as atraumatic as possible to ensure good dental rehabilitation [12]. Therefore, it is of paramount importance to pay attention to the bone and supporting tissues.

Not to forget the medically compromised patients, especially those with bleeding tendencies and those on antiplatelets, anticoagulants, or hemophiliac. Recent guidelines related to hemophiliac patients going for oromaxillofacial surgeries and European protocols, proposed by the European Society of Cardiology in the Focused Update on Dual Antiplatelet Therapy (DAPT), suggest a preoperative evaluation of risks of cardiac events in relation to the surgical procedure, such as bleeding, duration of surgery, and related stress [13, 14].

Over the years, surgeons realized the drawback of paying less attention to the surrounding alveolar complex structures. This realization was the stimulus for the development of the concept of minimally invasive extraction techniques. With the concomitant evolution of the newer dental implants, surgeons were focusing more on the preservation of dentoalveolar hard and soft tissue complex during extractions. In order to aid this atraumatic extraction approach, a variety of instruments came into the surgeons' aid, such as Benex vertical extractor, piezosurgery, periotome, and physics forceps, to name a few. The use of these instruments was to facilitate an atraumatic extraction as much as possible, both for the patient and the surrounding dentoalveolar structures [15]. These have greatly improved the predictability and reduced the invasiveness of oral surgery cases, especially with third molar impaction cases [16].

One of the instruments that was designed to facilitate an atraumatic extraction was the periotome. These instruments came into being about twenty-five years ago with the intention to enhance the luxation of the teeth and as an adjunct to conventional elevators [17]. The periotomes were designed to have fine and delicate working tips that could insert into the narrow periodontal ligament (PDL) space vertically along the long axis of the teeth. The design also allowed delivering mild vertical pressure to aid luxation [18, 19].

Although these instruments have their own drawback, periotomes have come to stay in routine dental extractions, especially with the beginning of newer dental implants. They have proven to reduce trauma to the soft and hard tissue and thereby maintaining the bony architecture [20].

With the rapid revolutionary advancement in technology, a new device has come to the market, with the claim of reducing time and atraumatic to the surrounding tissues during surgical procedures, which is the mechanical/automated periotome. Therefore, the objective of this study is to compare the automated periotome with a standard elevator with regard to operating time, postoperative gingival laceration, bone fracture, and tooth structures fractures.

## 2. Material and Method

The study is an in vitro cross-sectional observational study. Ethical approval was obtained (removed for blind peer review). The study took place in a closed dental unit (removed for blind peer review) with utmost infection control measures.

**Figure 1:**
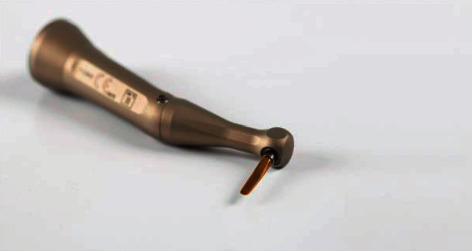
Luxator LX.

The materials used for this study were conventional periotomes along with mucoperiosteal elevator and lower premolar forceps, which are the commonly used instruments by surgeons in their daily practice, and a mechanical periotome (Luxator LX, Directa AB, SE-194 27 Upplands Vӓsby, Sweden). The latter's tip will only cut under pressure and is coated with titanium which makes it durable and remains sharp even after going through many sterilization cycles. In addition, the shape of the handpiece itself is contra-angled, which will make it easier to be used in the posterior region where less accessibility is encountered (Figure 1).

Sheep mandibles that were intact and not diseased or fractured (no visible overgrowth or cracks), fresh within 24 hours, and having sound teeth were included in the study. Jaws that were visibly damaged or diseased or not fresh and having defective teeth were not selected for the study. All bones were put in a preserving medium of an equal amount of 70% ethyl alcohol and saline. In addition, one operator extracted all teeth.

Ten sound sheep mandibles with two posterior teeth in each quadrant were used. Each jaw was divided into two groups to compare the automated periotome with the conventional periotome in terms of operating time, postoperative gingival laceration, bone fracture, and tooth structure fractures. Out of the forty teeth, twenty were extracted by an automated periotome while the other twenty by the conventional periotome. Each quadrant was tested by a separate device, and the reading was noted down and compared later.

**Figure 2:**
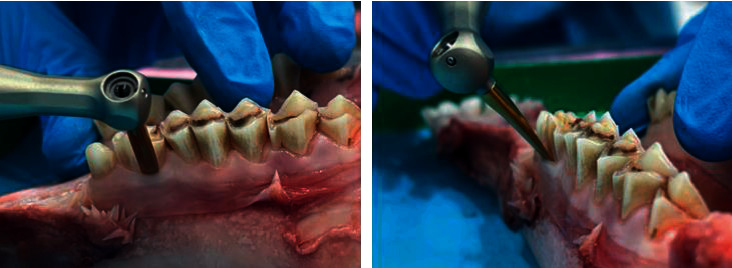
Extraction using the Luxator LX.

At the time of the procedure, the jaws were removed from the preserving medium and then placed on the working bench, which was covered with dental napkins. Two premolar teeth from each quadrant were extracted and their results were compared (20 by the conventional and 20 by the automated) (Figure 2).

The extraction started with the conventional periotome group. Once the operator started the mucoperiosteal detachment to detach the gingiva, the assistant started the timer and took notes. Upon completing the use with the mucoperiosteal elevator, the operator started placing the conventional periotome. The periotome blade was inserted into the gingival sulcus at an angle to the vertical axis of the tooth in order to first sever the cervical gingival attachment fibres. The angulation of the blade was adjusted depending on the vertical access of the tooth. In all cases, the blade should be on the root surface vertically. Later, the blade was gently inserted further into the narrow PDL space first on the mesial side and then on the distal side. Once access was obtained into the PDL space, the blade was advanced in the same motion into the PDL space until two-thirds of the root surface was reached. Finally, the tooth was extracted using extraction forceps. The extraction time was noted as soon as the tooth was out of the socket.

In the automated periotome group, we attached the contra-angle hand piece with the low-speed hand piece motor. The motor was set at 1000 to 4000 rpms. The assistant started the timer when the operator began detaching the gingiva with the mucoperiosteal elevator. Once the operator finished detaching the gingiva, he started using the mechanical luxator by placing it in a vertical angulation with the long access of the tooth to make sure the tip is between the bone and the tooth. The self-directing tip will start to follow the root surface once the operator moves it. The tip is moved in a reciprocating motion, up and down, cutting all the PDL fibres to two-thirds of the tooth. The tooth was luxated and removed with the extraction forceps. The extraction time was noted as soon as the tooth was out of the socket.

The assistant assessed the gingiva, bone, and tooth once the tooth was removed from the socket. Any gingival laceration, bone fractures or visible cracks, and tooth or root fractures were noted as “YES” or “NO.”

All data were analyzed using the Statistical Package for Social Science (SPSS, version 20.0.0 for Windows). The operating time was analyzed using the one-way ANOVA test after checking the normality of the data with the Shapiro–Wilk test. For the other parameters, the data are discrete and hence we used the Mann-Whitney test with the *P* value set at below 0.05 with a 95% confidence interval.

## 3. Results

Forty posterior teeth (premolars) were divided equally into two groups to compare automated periotome and conventional periotome in simple intra-alveolar extraction of ten sheep mandible jaw.

The study aimed to evaluate the operating time, postoperative gingival laceration, and bone, crown, and root fractures between the two groups.

**Figure 3:**
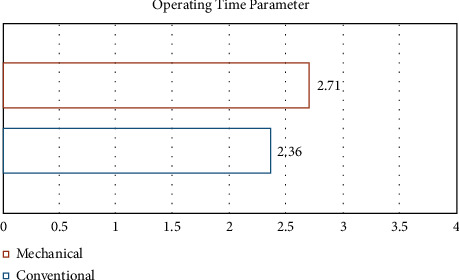
Operating time parameter.

The data were normally distributed with a 0.175 significance obtained from the Shaprio–Wilk test. One-way ANOVA was used for the operating time parameter, as it is continuous data. It shows that the automated periotome took a marginally longer time in extracting the premolars of the sheep mandible than the conventional periotome with a mean of 2.71 ± 1.93 and 2.36 ± 1.42, respectively. This did not show any statistical difference amongst the different groups (*P* < 0.856) (Figure 3).

For the other parameters, the Mann–Whitney test was used to obtain the *P* value between the groups. Three out of the twenty cases had gingival laceration with the automated periotome, as opposed to six out of the other twenty cases with the conventional periotome. Again, this did not show any statistical difference amongst the different groups (*P* < 0.262).

Secondly, the sheep mandible was evaluated for visible fractures in the bone. We found no fractures in extractions with automated periotome in all twenty cases, while noticed seven cases with bone fractures in extractions with conventional periotome, statistically with a *P* < 0.004.

Lastly, the extracted teeth were assessed for cracks or fractures in the crown or roots. The results showed no fractures of any of the crowns of all forty teeth. Two cases had a fracture of the tooth's root with the automated periotome and a single case with the conventional periotome. Statistically, no significant variance was seen between the two groups (*P* < 0.553) (Table 1).

## 4. Discussion

The modern oral surgical technique is moving away from the traditional extraction techniques of conventional dental elevators [21–23]. Atraumatic dental extractions are preferred nowadays, especially when dental implants are being considered. A number of techniques and instrument designs are proposed for accomplishing atraumatic extractions and one of the proposed instruments for achieving that is the periotome [24].

The periotome is an invaluable instrument in the armamentarium of any surgeons during an atraumatic extraction. This instrument helps in extracting teeth and remaining root stumps roots without injuring the surrounding housing and causes minimal or no laceration of the soft tissue. This helps in a better postoperative dental rehabilitation of the patients, including dental implant insertion. Thus, it supports the biomechanical justification for the minimally invasive technique of dental extraction [25]. In addition, a periotome is useful in extracting difficult teeth such as the endodontically treated teeth and crown fracture cases by maintaining soft and hard tissue architecture without the need for reflecting a flap and its postoperative consequences. Extractions with periotome result in an intact alveolus and near-normal extraction socket [18].

As an alternative, an automated periotome is an exciting technological advance in atraumatic extraction technique that is revolutionizing the field of dentistry. This device allows precise and atraumatic extraction of teeth in a short time, which means preservation of bone and gingival architecture and facilitates in placing immediate implants quicker. An automated periotome is an electrical device that encompasses a hand piece with a periotome that is controlled by foot control. In addition, it contains a microprocessor that removes uncertainty while extracting a tooth. The automated periotomes work on the mechanism of “wedging” and “severing” to facilitate tooth extraction. The main mechanical action is that the blade should conform with the anatomy of the roots in an apical direction with an increment of 2 to 3 mm [25].

In this study, the Luxator® LX automated periotome shows not much difference from the conventional periotome regarding the operating time, postoperative gingival laceration, and crown or root fractures. However, in terms of bone fractures, there is an advantage of using the automated periotome over the conventional periotome. This is an important factor for preserving good alveolar bone. Since implant dentistry is precision-based and measurements of millimeters in the bony architecture are vital to success, the preservation of the alveolar architecture improves the functional and cosmetic outcomes of dental rehabilitation [25].

White et al. in 2009 did a clinical study about the automated periotome. They had seven cases of dental extraction performed with the Powertome automated periotome without making a flap. They found out that in none of the cases, there was any damage to the dentoalveolar complex. They were able to perform most of the cases quickly. Their conclusion was that the automated periotome was a very practical and efficient instrument for performing atraumatic dental extractions. They also added the advantage of avoiding a mucoperiosteal flap reflection and the concomitant damage to the soft and hard tissue, especially the thin gingival papilla. All this eventually improves the insertion of an immediate or delayed dental implant [26].

James et al. in October 2019 did a preliminary study on the Powertome periotome. They performed fourteen teeth extraction, eight posterior and six anterior. The average of these extractions was 4.8 minutes. They also concluded that the automated periotome was not time-consuming in the dental extraction of single or multirooted teeth. In their opinion, the hand-operated periotomes (conventional periotomes) were less effective with the extraction of multirooted teeth and took more operating time [27].

Sharma et al. in 2015 evaluated the efficiency of the conventional periotomes in the nonsurgical extraction of single-rooted teeth. They did a randomized controlled study for one hundred patients that required an extraction for a single-rooted tooth. Their results showed that there was a significant pain reduction. Therefore, they conclude that the periotome might be suggested to reduce the postextraction discomfort [18]. Pain and postextraction discomfort parameters were impossible to add to our study since we performed the study on freshly cut sheep mandibles.

The drawbacks of the automated periotome such as the high cost of the armamentarium and the maintenance may put off clinicians from investing in this equipment. To our knowledge, there are no randomized controlled studies done with regard to this topic. The results of this in vitro study are encouraging. However, randomized controlled studies are essential to determine the effectiveness and advantage of these extraction techniques and instruments.

## 5. Conclusion

Regardless of the difference in anatomy and structures of the sheep teeth, we can conclude that both groups showed excellent outcomes. Fewer cases showed gingival laceration of the automated periotome than the conventional periotome, while the opposite was in terms of root fracture and duration of the procedure. Yet, all of the parameters have shown no statistically significant difference between the groups except for the bone fracture parameter, in which statistically, the automated periotome showed its benefits over the conventional periotome. In view of an immediate or future insertion of the dental implant, the preservation of the alveolar bone is a very important factor. Hence, it is worthwhile to use mechanical periotome in extractions especially when implants are considered in the treatment plan.

## Figures and Tables

**Table 1:** Comparison between the conventional and mechanical periotome in relation to gingival laceration, bone fracture, and crown and root fractures.

	Conventional periotome		Mechanical periotome		*P* value
	Yes	No	Yes	No	
Gingival laceration	6	14	3	17	0.262
Bone fracture	7	14	0	20	0.004^*∗*^
Crown fracture	0	20	0	20	1
Root fracture	2	18	1	19	0.553
Total	20		20		

^*∗*^Significant difference.

## Data Availability

All the data related to the article are available with the corresponding and submitting authors.
